# Factors affecting duration of stay in the intensive care unit after coronary artery bypass surgery and its impact on in-hospital mortality: a retrospective study

**DOI:** 10.1186/s13019-024-02527-y

**Published:** 2024-02-03

**Authors:** Khalid S. Ibrahim, Khalid A. Kheirallah, Abdel Rahman A. Al Manasra, Mahmoud A. Megdadi

**Affiliations:** 1 Division of Cardiac Surgery, Department of General Surgery and Urology, Faculty of Medicine, Jordan University of Science and Technology and Princess Muna Heart Center, King Abdullah University Hospital, Irbid, Jordan; 2 Department of Public Health and Community Health, Faculty of Medicine, Jordan University of Science and Technology, Irbid, Jordan; 3 Department of General Surgery and Urology, Faculty of Medicine, Jordan University of Science and Technology, Irbid, Jordan

**Keywords:** CABG surgery, Prolonged ICU stay, Ventilator support, Pneumonia, Cost

## Abstract

**Background:**

Different risk factors affect the intensive care unit (ICU) stay after cardiac surgery. This study aimed to evaluate these risk factors.

**Patients and methods:**

A retrospective analysis was conducted on clinical, operative, and outcome data from 1070 patients (mean age: 59 ± 9.8 years) who underwent isolated coronary bypass grafting CABG surgery with cardiopulmonary bypass. The outcome variable was prolonged length of stay LOS in the CICU stay (> 3 nights after CABG).

**Results:**

Univariate predictors of prolonged ICU stays included a left atrial diameter of > 4 cm (*P* < 0.001),chronic obstructive airway disease COPD (*P* = 0.005), hypertension (*P* = 0.006), diabetes mellitus (*P* = 0.009), having coronary stents (*P* = 0.006), B-blockers use before surgery (either because the surgery was done on urgent or emergency basis or the patients have contraindication to B-blockers use) (*P* = 0.005), receiving blood transfusion during surgery (*P* = 0.001), post-operative acute kidney injury (AKI) (*P* < 0.001), prolonged inotropic support of > 12 h (*P* < 0.001), and ventilation support of > 12 h (*P* < 0.001), post-operative sepsis or pneumonia (*P* < 0.001), post-operative stroke/TIA (*P* = 0.001), sternal wound infection (*P* = 0.002), and postoperative atrial fibrillation POAF (*P* < 0.001).

Multivariate regression revealed that patients with anleft atrial LA diameter of > 4 cm (AOR 2.531, *P* = 0.003), patients who did not take B-blockers before surgery (AOR 1.1 *P* = 0.011), patients on ventilation support > 12 h (AOR 3.931, *P* =  < 0.001), patients who developed pneumonia (AOR 20.363, *P* =  < 0.001), and patients who developed post-operative atrial fibrillation (AOR 30.683, *P* =  < 0.001) were more likely to stay in the ICU for > 3 nights after CABG.

**Conclusion:**

Our results showed that LA diameter > 4 cm, patients who did not take beta-blockers before surgery, on ventilation support > 12 h, developed pneumonia post-operatively, and developed POAF were more likely to have stays lasting > 3 nights. Efforts should be directed toward reducing these postoperative complications to shorten the duration of CICU stay, thereby reducing costs and improving bed availability.

## Background

Coronary artery bypass grafting (CABG) is the most commonly performed type of cardiac surgery worldwide. CABG aims to improve the quality of life of patients by alleviating angina and heart failure symptoms and increasing survival rates [1]. However, CABG is associated with risk of morbidity and mortality [2]. Previous studies reported mortality rates after an isolated CABG ranging from 2.6 to 12.2% [3–5]. Predictors of post-surgical mortality and morbidity have been studied thoroughly in various countries [3–6]. In Jordan, we previously studied the determinants of three complications after isolated CABG surgery (renal impairment, pneumonia, and sternal wound infection). We found that age, female sex, history of diabetes mellitus, COPD, peripheral vascular disease, renal impairment, emergency surgery, perioperative blood transfusion, mechanical ventilation of > 12 h, and prolonged inotropic support were associated with 30-day complications after on-pump isolated CABG surgery [6].

Cardiac intensive care units (CICUs) are specialized units that provide care to patients after cardiac surgery or critically ill patients. However, the care provided by intensive care units is costly and labor-intensive. Compounded by a limited number of ICU beds, most ICUs operate at or near full capacity [7]. Thus, bed unavailability has become a critical issue that can significantly impact other services, including operating theatres. Extending ICU capacity may not always be feasible due to physical constrains, resources limitations, or government regulations [8]. For these reasons, the duration of ICU stay is of major importance following cardiac surgery, as well as other types of surgery.

Perioperative management of patients undergoing (CABG) has been improving recently, but the duration of LOS in the CICU stay varies from one to several days for various reasons. Prolonged LOS in the CICU stay in these patients increases both overall hospital and CICU costs [9] and reduces the availability of CICU beds for other critically ill patients. Therefore, the ability to predict the duration of CICU stay in patients undergoing CABG is invaluable. Many studies [10, 11] have reported perioperative risk factors for prolonged ICU stay, with prolonged mechanical ventilation considered one of the most critical factors. Other studies have indicated that prolonged inotrope use and blood transfusion in the CICU can predict the duration of LOS [12].

In this study, we assessed the factors influencing the length of ICU stay in patients following CABG by analyzing pre-operative, intra-operative, and immediate postoperative variables.

## Patients and methods

This retrospective cohort study included all patients who underwent isolated CABG at the Princess Mona Heart Institute/King Abdullah University Hospital in northern Jordan between January 2005 and June 2022. This study was approved by the Institutional Review Board of King Abdullah University Hospital. The exclusion criteria included repeat surgery, valve surgery, and combined CABG and valve surgery. A total of 1070 consecutive patients were included in this study. Patients are typically transferred to the CICU immediately after surgery. The CICU, specializing in cardiac surgery, is a well-equipped unit with six beds and a patient to nurse ratio of 1:1. Patients were transferred to the ward once they achieved hemodynamic stability, were successfully weaned from the ventilator, were able to ambulate, and no longer required inotropes and vasopressors. This transfer occurred either until the removal of chest drains or their output remained consistently low (5–6 consecutive hours with no drainage). The criteria for weaning from the ventilator included hemodynamic stability, adequate urine output, regaining full consciousness (including obeying commands), minimal chest tube drainage (< 50 cc/hour), normal chest X-ray findings, and normal arterial blood gases on continuous positive airway pressure CPAP and T-piece. All patients were transferred from the CICU after ensuring that they did not require reexploration for bleeding. Staying for ≤ 3 nights in the CICU was considered normal, whereas staying for > 3 nights was considered prolonged. The average stay of three nights struck a balance between patient stabilization, cost consideration, and limited resources of the CICU. In this study, patients were categorized into two groups: normal (n = 276) and prolonged (n = 794) stays. Of the patients with prolonged ICU stay, clinical, laboratory, and demographic data were obtained retrospectively from the patients’ medical records. Data on plasma glucose, low-density lipoprotein, high-density lipoprotein, triglyceride, and total cholesterol levels were obtained from the King Abdullah University Hospital Information Registry. Preoperative medication use was collected from all the patient data and assessed in relation to the duration of ICU stay following CABG.

Myocardial infarction (MI) was defined according to the fourth universal definition of MI. It entails an elevation or drop in cardiac enzymes (specifically troponin) beyond the 99th percentile of the upper reference limit, combined with at least one of the following: MI symptoms, new electrocardiogram (ECG) ischemic changes, the presence of pathological Q wave, or imaging evidence consistent with ischemia [13].

ST-elevation myocardial infarction STEMI was defined according to the European Society of Cardiology (ESC) guidelines: at least two contiguous leads with ST-segment elevation of ≥ 2.5 mm in men < 40 years, ≥ 2 mm in men ≥ 40 years, or ≥ 1.5 mm in women, particularly in leads V2–V3, and/or ≥ 1 mm in the other leads in the absence of left ventricular (LV) hypertrophy or left bundle branch block (LBBB) [14].

Recent myocardial infarction was defined as MI within 28 days after CABG. Heart failure was considered if the patient was symptomatic or undergoing anti-failure treatment. Diabetes status (taking either oral hypoglycemic agents or insulin), hypertension status (being on treatment), renal dysfunction (having creatinine serum level of 2.0 mg/dl or on chronic dialysis), post-operative acute kidney injury AKI(having serum creatinine doubled after surgery), COPD (having diagnosed by pulmonogist at any time before surgery), peripheral vascular disease (having a positive history of intermittent claudication or having a documented clinical or radiological evidence of ischemia) were all recorded. Left ventricular ejection fraction was obtained from transthoracic echocardiography measurements. Left ventricular ejection fraction (LVEF) was classified into three groups according to the degree of dysfunction: normal (LVEF ≥ 50%), mild to moderate impairment (LVEF between 36 and 49%), and severe impairment (LVEF ≤ 35%). The number of diseased coronary arteries was obtained from the coronary angiography report. Patients with significant coronary artery disease underwent CABG according to American College of Cardiology (ACC) and American Heart Association (AHA) guidelines [15–17]. Surgery was considered emergency or urgent if the patient was sent to the operating room within 24 h from the time of cardiac catheterization, typically due to unstable angina, hemodynamic instability, or an untoward event in the catheterization suite. Preoperative trans-thoracic echocardiography studies were performed to evaluate left ventricular ejection fraction LVEF, the degree of mitral/aortic/tricuspid valve regurgitation, and left atrial LA size (diameter). MR was graded from 0 to 4, with 0 = competent, 0–1 = trace MR, 1–2 = mild MR, 2–3 = moderate MR, and 3–4 = severe MR. Left atrial diameter was calculated using the long axis view, measuring the anteroposterior dimension in M-mode. Cardiopulmonary bypass time (pump time) was considered prolonged if > 120 min, and cross clamp time was considered prolonged if > 90 min. Inotropic support was considered prolonged if it continues for 48 h or more, and ventilation support was considered prolonged if it stayed > 12 h. Pneumonia was diagnosed based on symptoms (cough, fever, and SOB), and radiological or microbiological evidence was diagnosed from sputum culture. Sternal wound infection was defined based on presence of purulent discharge from the wound, positive wound culture, or radiological evidence of mediastinitis. Stroke was defined as a permanent neurological deficit by clinical examination and with radiological evidence by brain CT scan or MRI. Pre-, peri-, and postoperative measurements were evaluated as possible independent risk factors for prolonged ICU stay after CABG.

### Statistical analysis

SPSS version 22 was used for data analysis. Frequencies and percentages were used to summarize categorical variables, while mean ± standard deviation was used for continuous variables. Duration of ICU stay was dichotomized into ≤ 3 nights or > 3 nights. Independent sample t-tests or χ^2^ tests were used to analyze independent variables for prolonged ICU stay (> 3 days), as appropriate. P-values are reported for bivariate analyses. All independent variables that were significantly associated with a prolonged ICU stay (*P* < 0.05) were included in the backward conditional logistic regression model (entry at *P* = 0.05, removal at *P* = 0.2). Adjusted odds ratios (AOR) and *P* values are reported. The alpha level for all analyses was set at 0.05. A logistic regression model was used to include variables that were significantly associated with a prolonged ICU stay at the bivariate level. Variable collinearity was tested using a multicollinearity diagnostic test, and the VIF was inspected accordingly. None of the VIF values were greater than 1.2.

## Results

The mean age of the study participants was 59 years, and males comprised approximately three quarters of the cohort (77.8%). Approximately two-thirds of the participants had hypertension, half had diabetes mellitus, and only 12.1% had heart failure. Table 1 shows the main preoperative demographic and clinical characteristics, while Table 2 shows the intra- and postoperative demographic and clinical characteristics of the study participants (N = 1070), as well as the unadjusted risks of clinical variables on the duration of ICU stay. As expected for a CABG population, 80% had stable angina, while approximately 20% had experienced a recent (≤ 28 days) myocardial infarction. Most patients (76%, 794 patients) had LOS in the ICU for ≤ 3 nights, while about one-fourth of the participants had > 3 days of ICU stay (26%, 276 patients). Preoperatively, most patients received statins, B-blockers, clopidogrel, and angiotensin-converting enzyme inhibitors, while 27.3% received diuretics. Approximately 16% of patients reported stopping clopidogrel for at least a week before surgery. Most participants underwent 4–6 bypass grafts, from the left internal mammary artery to the left anterior descending artery used in the majority of patients. Extended pump time (≥ 120 min) and aortic clamp time (≥ 90 min) were documented in 15.5% and 7.2% of patients, respectively. Sternal wound infections and pneumonia were diagnosed in 3.3% and 5.1% of patients, respectively, while 6.0% of patients had an ICU stay of a week or more. The prevalence of postoperative AKI was 8.1%. Incidence of postoperative atrial fibrillation was documented in 25.5% of the patients. Of the patients’ category with prolonged stay in the CICU, only 20 were discharged and readmitted to the ICU due to arrhythmia (nine patients), hemodynamic instability (six patients), and wound infection (five patients), while in the early discharge group from the CICU, only six patients were readmitted due to atrial fibrillation (four patients) and hemodynamic instability (two patients).

**Table 1:** Distribution of Participants by Pre-operative variables and Background Characteristics

Variables		3 Nights or less stay in CICU after CABG		More than 3 nights stay in CICU after CABG		Total	*P*-value
		n	%	n	%		
Overall		793	74.1	276	25.8	1070	
Age in years (Mean)		59.31(9.88)		58.37 (9.57)			0.170
BMI (mean)		28.58 (5.12)		28.65 (4.52)			0.846
PCV (mean)		39.59- (5.63)		39.20 (5.61)			0.356
LA Diameter (mean)		3.81 (0.34)		3.91 (.40)			< 0.001
Gender	Female	171	72.2	66	27.8	237	0.418
	Male	622	74.8	210	25.2	832	
	Total	793	74.2	276	25.8	1069	
CABG							0.584
	CABG X1 + X2	78	72.2	30	27.8	108	
	CABG X3	273	76.7	83	23.3	356	
	CABG X4	321	72.6	121	27.4	442	
	CABG X5 + X6	107	74.3	37	25.7	144	
	Total	779	74.2	271	25.8	1050	
LIMA-LAD							
	NO	110	74.8	37	25.2	147	0.827
	YES	651	74.0	229	26.0	880	
	Total	761	74.1	266	25.9	1027	
Smoking							
Stable Angina	Never Smoked	369	73.2	135	26.8	504	0.986
	ever Smoked	359	73.3	131	26.7	490	
	Total	728	73.2	266	26.8	994	
	NO	151	71.6	60	28.4	211	0.341
	YES	640	74.8	216	25.2	856	
	Total	791	74.1	276	25.9	1067	
History of Myocardial Infarction	NO	558	74.7	189	25.3	747	0.571
	YES	233	73.0	86	27.0	319	
	Total	791	74.2	275	25.8	1066	
Recent MI (28 within days)	NO	642	73.7	229	26.3	871	0.481
	YES	150	76.1	47	23.9	197	
	Total	792	74.2	276	25.8	1068	
COPD	NO	772	74.9	259	25.1	1031	0.005
	YES	15	51.7	14	48.3	29	
	Total	787	74.2	273	25.8	1060	
Hypertension	NO	322	78.7	87	21.3	409	0.006
	YES	465	71.1	189	28.9	654	
	Total	787	74.0	276	26.0	1063	
Hyperlipidemia	NO	513	75.4	167	24.6	680	0.283
	YES	254	72.4	97	27.6	351	
	Total	767	74.4	264	25.6	1031	
Diabetes	NO	379	78.0	107	22.0	486	0.009
	YES	408	71.0	167	29.0	575	
	Total	787	74.2	274	25.8	1061	
History of HF	NO	703	75.0	234	25.0	937	0.065
	YES	87	67.4	42	32.6	129	
	Total	790	74.1	276	25.9	1066	
PVD	NO	750	73.7	267	26.3	1017	0.193
	YES	41	82.0	9	18.0	50	
	Total	791	74.1	276	25.9	1067	
Atrial. fibrillation	NO	788	74.2	274	25.8	1062	0.107
	YES	1	33.3	2	66.7	3	
	Total	789	74.1	276	25.9	1065	
Renal impairment	NO	690	74.5	236	25.5	926	0.480
	YES	64	78.0	18	22.0	82	
	Total	754	74.8	254	25.2	1008	
EF	EF = > 50%	409	76.4	126	23.6	535	0.079
	EF 49% -35%	243	70.2	103	29.8	346	
	EF = < 34%	56	69.1	25	30.9	81	
	Total	708	73.6	254	26.4	962	
Pre-operative coronary Stents	NO	713	75.5	231	24.5	944	0.006
	YES	58	62.4	35	37.6	93	
	Total	771	74.3	266	25.7	1037	
Emergency	Not emergency	575	75.3	189	24.7	764	0.344
	Emergency	210	72.4	80	27.6	290	
	Total	785	74.5	269	25.5	1054	
ACE inhibitors	No	335	73.3	122	26.7	457	0.623
	Less than mont	146	76.8	44	23.2	190	
	More than month	304	73.6	109	26.4	413	
	Total	785	74.1	275	25.9	1060	
B-blockers	No	200	67.6	96	32.4	296	0.005
	Less than month	206	79.2	54	20.8	260	
	More than month	379	75.2	125	24.8	504	
	Total	785	74.1	275	25.9	1060	
Statins	No	175	70.6	73	29.4	248	0.350
	Less than month	192	75.6	62	24.4	254	
	More than month	418	74.9	140	25.1	558	
	Total	785	74.1	275	25.9	1060	
Diuretics	No	583	75.7	187	24.3	770	0.013
	Less than month	62	62.0	38	38.0	100	
	More than month	139	73.9	49	26.1	188	
	Total	784	74.1	274	25.9	1058	
Plavix	No	287	74.9	96	25.1	383	0.709
	Discontinue for less than 7 days	379	74.2	132	25.8	511	
	Discontinue for more than 7 days	121	71.6	48	28.4	169	
	Total	787	74.0	276	26.0	1063	
MR	No	433	76.2	135	23.8	568	0.249
	MR GRAD 1	248	72.1	96	27.9	344	
	MR GRAD 2 + 3	43	69.4	19	30.6	62	
	Total	724	74.3	250	25.7	974	

*BMI* body mass index, *PCV* packed cell volume, *LA* left atrium, *CABG* coronary Arteru bypass grafting, *LIMA-LAD* Left internal mammary artery, *MI* Myocardial Infarction, *COPD* chronic obstructive pulmonary disease, *HF* heart failure, *PVD* perephral vascular disease, EF ejection fraction, *ACE* Angiotension Convertting Enzyme, *MR* mitral regurgitation

**Table 2:** Distribution of Participants by intra and post-operative Characteristics

Variables		3 Nights or less stay in CICU	(%)	More than 3 nights stay in CICU	(%)	*P*-value	
Pump time	Pump Time < 120 min	640	73.6	229	26.4	869	0.649
	Pump Time > 120 min	120	75.5	39	24.5	157	
	Total	760	73.9	268	26.1	1028	
Aorta cross- clamp	Aorta. Clamp < 90 min	709	73.9	250	26.1	959	0.910
	Aorta. Clamp > 90 min	55	73.3	20	26.7	75	
	Total	764	73.9	270	26.1	1034	
Intra-operative blood transfusion	No	184	66.4	93	33.6	277	0.001
	Yes	590	76.6	180	23.4	770	
	Total	774	73.9	273	26.1	1047	
Re-exploration	No	703	74.3	243	25.7	946	0.591
	Yes	66	71.7	26	28.3	92	
	Total	769	74.1	269	25.9	1038	
Prolonged inotropic support	less than 12 hs	485	77.1	144	22.9	629	< 0.001
	12–24 hs	104	71.7	41	28.3	145	
	24–36 hs	44	77.2	13	22.8	57	
	36–48	89	80.2	22	19.8	111	
	48–72	41	71.9	16	28.1	57	
	More than 72 hs	2	5.4	35	94.6	37	
	Total	765	73.8	271	26.2	1036	
Ventilation duration	Vent. Duration < 12Hrs	624	80.7	149	19.3	773	< 0.001
	Vent. Duration > 12Hrs	147	55.9	116	44.1	263	
	Total	771	74.4	265	25.6	1036	
Postoperative AKI	No	740	76.7	225	23.3	965	< 0.001
	Yes	53	51.0	51	49.0	104	
	Total	793	74.2	276	25.8	1069	
Pneumonia/sepsis	No	775	77.2	229	22.8	1004	< 0.001
	Yes	6	11.8	45	88.2	51	
	Total	783	74.1	274	25.9	1057	
Postoperative stroke/TIA	No	781	74.7	265	25.3	1046	0.001
	Yes	3	30.0	7	70.0	10	
	Total	784	74.2	272	25.8	1056	
Sternal infection	No	764	74.8	258	25.2	1022	0.002
	Yes	18	51.4	17	48.6	35	
	Total	782	74.0	275	26.0	1057	
Post-operative AF	No	736	90.2	80	9.8	816	< 0.001
	Yes	54	21.7	195	78.3	249	
	Total	790	74.2	275	25.8%	1065	

*CICU* cardiac intensive care unit, *TIA* transient ischemic attack, *AF* Atrial fibrillation, *AKI* Acute kidney injury

Overall, 60 patients (5.6%) died within 30 days. Most mortalities occurred in those who stayed for > 3 nights. Among those who stayed for > 3 nights, mortality rate was 12.5%, compared to 4.8% among those who reported staying ≤ 3 nights (*P* < 0.001). Those who reported to stay > 3 nights were 2.87 times as likely to die compared to those who stayed ≤ 3 nights (95% CI 1.78–4.65).

Mean (SD) ICU stay was 3.34 (2.05) nights with a range between 0 and 23 nights (median and IQR are 3.00 and 2.00, respectively). More than one quarter of participants (25.8%, n = 276) had an ICU stay of > 3 nights.

Univariate predictors of prolonged ICU stay included left atrial diameter > 4 cm, COPD, hypertension, diabetes mellitus, history of coronary stents, beta-blockers use before surgery, receiving blood transfusion during surgery, postoperative AKI, prolonged inotropic support for > 12 h, ventilation support for > 12 h, postoperative sepsis or pneumonia, post-operative stroke/TIA, sternal wound infection, and postoperative atrial fibrillation.

The multivariate logistic regression model included all variables that were associated with postoperative prolonged ICU stay (*P* < 0.2), along with variables of clinical significance: age, sex, and body mass index (Table 3). Patients with LA diameter > 4 cm (AOR 2.531, *P* = 0.003), patients who did not take beta-blockers before surgery (AOR 1.1, 2.7, *P* = 0.011), patients on ventilation support > 12 h (AOR 3.931, *P* =  < 0.001), patients who developed pneumonia (AOR 20.363, *P* =  < 0.001), and patients who developed post-operative atrial fibrillation (AOR 30.683, *P* =  < 0.001) were more likely to stay for > 3 nights in the ICU after CABG.

**Table 3:** Adjusted effect of selected variables on perioperative length of stay (Backword—Conditional)

Variables		3 Nights or less stay in CICU		More than 3 nights stay in CICU		Total	OR	*P*-value
		n	%	n	%			
Overall		793	74.1	276	25.8	1070		
LA diameter		3.81		3.91			2.531	0.003
Hypertension	NO	322	78.7	87	21.3	409	1.525	0.092
	YES	465	71.1	189	28.9	654		
	Total	787	74.0	276	26.0	1063		
PVD	NO	750	73.7	267	26.3	1017	0.232	0.049
	YES	41	82.0	9	18.0	50		
	Total	791	74.1	276	25.9	1067		
Atrial fibrillation	NO	788	74.2	274	25.8	1062	24.884	0.012
	YES	1	33.3	2	66.7	3		
	Total	789	74.1	276	25.9	1065		
B-Blockers	No	200	67.6	96	32.4	296	Ref	0.011
	Less than month	206	79.2	54	20.8	260	0.360	
	More than month	379	75.2	125	24.8	504	0.903	
	Total	785	74.1	275	25.9	1060		
Diuretics	No	583	75.7	187	24.3	770	Ref	0.087
	Less than month	62	62.0	38	38.0	100	2.374	
	More than month	139	73.9	49	26.1	188	0.808	
	Total	784	74.1	274	25.9	1058		
Prolonged support	Less than 12 hs	485	77.1	144	22.9	629	Ref	0.150
	12–24 hs	104	71.7	41	28.3	145	0.677	
	24–36 hs	44	77.2	13	22.8	57	0.605	
	36–48	89	80.2	22	19.8	111	0.616	
	48–72	41	71.9	16	28.1	57	0.699	
	more than 72 hs	2	5.4	35	94.6	37	0.008	
	Total	765	73.8	271	26.2	1036		
Ventilation duration	Vent. Duration < 12Hrs	624	80.7	149	19.3	773	3.931	0.000
	Vent. Duration > 12Hrs	147	55.9	116	44.1	263		
	Total	771	74.4	265	25.6	1036		
Pneumonia/sepsis	No	775	77.2	229	22.8	1004	20.363	0.000
	Yes	6	11.8	45	88.2	51		
	Total	783	74.1	274	25.9	1057		
Postoperative AF	No	736	90.2	80	9.8	816	30.683	0.000
	Yes	54	21.7	195	78.3	249		
	Total	790	74.2	275	25.8	1065		

*LA* left atrium, *PVD* peripheral vascular disease, *AF* atrial fibrillation

## Discussion

The treatment process for cardiac surgery patients is complex and involves pre-, intra-, and postoperative care provided by different multidisciplinary teams at each stage. The cardiac surgeon who performs the operation should serve as the central figure responsible for coordinating and connecting the various stages of the process. Postoperative complications, which influence hospitalization in the ICU, are the results of events occurring at all three perioperative stages; frequently, the ICU is the last stage, where shortcomings and errors of earlier stages, which determine the length of ICU stay, guarantee the quality of the result of cardiac surgery.

In this study, we present our low-volume, single-center experience of the duration of CICU stays after CABG at our center. Pre-, intra-, and postoperative variables were included in a multivariate regression model to predict duration of ICU stay in 1070 patients who underwent isolated CABG surgery.

Advanced age has been shown in some studies to predict prolonged ICU stay and other morbidities after CABG and other cardiac surgeries [6, 9]. In this study, age was not a predictor of prolonged stay after isolated CABG. We believe that this is related to the fact that our cohort was relatively young (mean age, 59 years).

We have previously reported a strong association between left atrial enlargement and mortality after isolated CABG and valve surgery [18, 19]. Our study also showed an association between left atrial enlargement and prolonged ICU stay. Other reports have also shown a relationship between LA size and prolonged ICU stay [20]. LA enlargement may indicate poor LV function and disease chronicity, particularly mitral valve disease.

Borzak et al. [21] showed that post-operative atrial fibrillation (POAF) is an independent predictor of prolonged ICU stay independent of advanced age. Our finding that POAF is an independent predictor of prolonged ICU stay confirms that POAF by itself is a strong predictor of prolonged ICU stay after CABG. In our CICU, POAF is considered as an indication for keeping patients in the unit.

Ventilator support of > 12 h was shown to be a predictor of prolonged ICU stay after isolated CABG in this study cohort. Many studies [22, 23] have published similar results. Prolonged ventilatory support may reflect poor lung function before surgery or a complicated course intra- or postoperatively.

Despite numerous preventive measures, pneumonia remains the most common major infection after cardiac surgery. It is associated with high mortality and morbidity and length of stay in the ICU as evidenced in many studies [24]. In this study, postoperative pneumonia was shown to strongly predict postoperative ICU stay. Pneumonia may prolong the time of ventilation or even mandate reintubation of these patients, which may prolong their stay in the CICU.

In this study, patients who did not use beta-blockers before surgery stayed in the ICU longer than those who used them preoperatively. This might be due to the fact that B-blockers decrease POAF [25] which, as discussed earlier, can prolong stay in the ICU after CABG. Patients who did not use beta-blockers were those who underwent urgent or emergency surgery with no time period between diagnosis and surgery; therefore, the prolongation might be due to this factor. In our ICU, beta-blockers were started or resumed the day after surgery in all patients, except those who were on beta-agonist inotropes.

Female gender was found by some researches to be a risk factor for prolonged stay in the CICU after CABG [26]. In our study, this result was not repeated. It could be due to differences in risk factors for this cohort.

Hypertension, DM, dyslipidemia and smoking [27, 28] have not been associated with a prolonged LOS in patients undergoing CABG, which was also observed in our results. Other study [26] found that smoking is a predictor of prolonged stay in the CICU.

Through its activating effect on the complement system and release of cytokines, blood transfusions can cause lung problems and increase the incidence of lung infection and so increases the mortality and morbidity rates and consequently the length of CICU stay after CABG. Cardiopulmonary bypass machine is known to reduce total peripheral resistance specially if used for long times which usually needs prolonged use of vasopressors to treat it. This adds to the duration of stay in the ICU after cardiac surgeries [12]. In our study, neither intraoperative blood transfusion nor prolonged CPB time were associated with prolonged stay in the CICU.

Studying the factors that affect the length of stay in the ICU aims to highlight the importance of reducing or eliminating them to reduce the duration of ICU stay, thus reducing costs and increasing the availability of beds for patients who need them.

### Limitations of the study

This was a retrospective study. It was done over a long time and by many surgeons in a relatively small sized center, all these limitations make drawing solid universal conclusions difficult. However, we have presented our center’s results and, to our knowledge, this is the first report from Jordan to discuss this issue. A larger prospective multicenter study is required to confirm these findings.

## Conclusion

CICUs management is a continuation of pre- and intraoperative care management, and the length of stay is usually determined by complications that reflect events occurring in the pre- or intra- operative era. Our results showed that LA diameter > 4 cm, patients who did not take beta-blockers before, patients on ventilation support > 12 h, patients who developed pneumonia postoperatively, and patients who developed post-operative atrial fibrillation were more likely to stay for > 3 nights in the ICU after CABG. Efforts should be made to reduce these postoperative complications to reduce the duration of ICU stay, thereby reducing costs and improving bed availability.

## Data Availability

Available upon request.

## Abbreviations

CABG: Coronary artery bypass grafting
ICU: Intensive care unit
AKI: Acute kidney injury
BMI: Body mass index
LA: Left atrium
AOR: Adjusted odds ratio
HF: Heart failure
EF: Ejection fraction
LVEF: Left ventricular ejection fraction
LIMA: Left internal mammary artery
LAD: Left anterior descending
CAD: Coronary artery disease
ACE-i: Angiotensin-converting enzyme inhibitor
ET-1: Endothelin 1
IRB: Institutional Research Board
